# Draft Genome Sequences of Eight Nontypeable *Haemophilus influenzae* Strains Previously Characterized Using an Electrophoretic Typing Scheme

**DOI:** 10.1128/genomeA.01374-15

**Published:** 2015-11-25

**Authors:** Huda J. Mussa, Timothy M. VanWagoner, Daniel J. Morton, Thomas W. Seale, Paul W. Whitby, Terrence L. Stull

**Affiliations:** a Department of Pediatrics, University of Oklahoma Health Sciences Center, Oklahoma City, Oklahoma, USA; b Department of Microbiology and Immunology, University of Oklahoma Health Sciences Center, Oklahoma City, Oklahoma, USA

## Abstract

Nontypeable *Haemophilus influenzae* is an important cause of human disease. Strains were selected for genome sequencing to represent the breadth of nontypeable strains within the species, as previously defined by the electrophoretic mobility of 16 metabolic enzymes.

## GENOME ANNOUNCEMENT

Nontypeable *Haemophilus influenzae* (NTHi) colonizes the human nasopharynx and is an opportunistic pathogen causing significant human disease. NTHi causes both mucosal and invasive infections, including otitis media, exacerbations of chronic obstructive pulmonary disease, and bacteremia (1–3). No vaccine currently exists to prevent NTHi colonization and/or infection. One essential characteristic of potential protective antigens is that they be broadly distributed across the target species. To facilitate ongoing studies to identify potential vaccine components, we genomically sequenced selected NTHi isolates from a collection previously characterized by the electrophoretic mobility of 16 metabolic enzymes and presumed to represent the breadth of the species (4, 5).

We performed whole-genome sequencing on the 8 selected isolates. NTHi samples were prepared using 50 ng of total genomic DNA, according to Nextera DNA library kit protocols (Illumina, Inc). Samples were indexed according to standard protocols so that they could be pooled together and sequenced simultaneously in a single run on the Illumina MiSeq using paired-end 150-bp (300-cycle; strains HI1373, HI1374, HI1388, HI1394, HI1408, HI1417, and HI1426) or paired-end 250-bp (500-cycle; strain HI1413) chemistry. Prior to sequencing, all libraries were run individually on the Agilent high-sensitivity DNA chip to confirm library quality and average insert size. Samples were pooled in equimolar amounts, and 8 pM the pool was run on the sequencer. Per Illumina’s recommendation, a PhiX control was spiked into the library pool prior to loading for quality control purposes. For the 300-cycle runs, 5 to 10% PhiX was used, and for the 500-cycle run, 1% PhiX was used, as per the Illumina protocols. Thirty to forty million reads in total were collected for each run. Raw sequence data were aligned to a reference isolate using CLC Genomics Workbench (CLC bio) to identify single-nucleotide polymorphisms (SNPs), and regions with insertions or deletions (indels) or raw data files were assembled using CLC *de novo* assembly parameters. The sequencing results are summarized in Table 1. The sequences reported here will facilitate further studies of NTHi disease to determine broadly distributed virulence determinants and vaccine candidates.

**TABLE 1:** Summary of genome sequences for eight selected NTHi isolates

Strain name	Source	BioSample no.	Accession no.	Genome coverage (×)	No. of contigs	G+C content (%)	Genome size (Mb)	No. of genes	ET^a^
HI1373	Ear	SAMN03702698	LFDP00000000	193	24	38.0	1.84	1,840	13
HI1374	CSF^b^	SAMN03702699	LFDO00000000	430	30	38.0	1.86	1,843	26
HI1388	Ear	SAMN03702700	LFDN00000000	588	16	37.8	1.81	1,770	43
HI1394	Ear	SAMN03702701	LFDM00000000	519	21	37.9	1.78	1,718	53
HI1408	CSF	SAMN03702702	LFDJ00000000	635	27	38.0	1.90	1,892	68
HI1413	Ear	SAMN03840566	LHSM00000000	372	54	38.2	1.95	1,972	73
HI1417	Blood	SAMN03702703	LFDK00000000	458	15	37.9	1.83	1,784	77
HI1426	CSF	SAMN03702704	LFDL00000000	575	18	38.0	1.85	1,831	86

a ET, electrophoretic type (5).

b CSF, cerebrospinal fluid.

### Nucleotide sequence accession numbers.

Genome sequences for the eight strains described here have been deposited in GenBank under the accession numbers listed in Table 1.
